# Serodiversity of Opsonic Antibodies against *Enterococcus faecalis* —Glycans of the Cell Wall Revisited

**DOI:** 10.1371/journal.pone.0017839

**Published:** 2011-03-18

**Authors:** Christian Theilacker, Zbigniew Kaczyński, Andrea Kropec, Irina Sava, Libin Ye, Anna Bychowska, Otto Holst, Johannes Huebner

**Affiliations:** 1 Center for Infectious Disease and Travel Medicine and Center for Chronic Immunodeficiency, University Medical Center Freiburg, Freiburg, Germany; 2 Faculty of Chemistry, University of Gdansk, Gdansk, Poland; 3 Division of Structural Biochemistry, Research Center Borstel, Leibniz-Center for Medicine and Biosciences, Borstel, Germany; University of Massachusetts Medical Center, United States of America

## Abstract

In a typing system based on opsonic antibodies against carbohydrate antigens of the cell envelope, 60% of *Enterococcus faecalis* strains can be assigned to one of four serotypes (CPS-A to CPS-D). The structural basis for enterococcal serotypes, however, is still incompletely understood. Here we demonstrate that antibodies raised against lipoteichoic acid (LTA) from a CPS-A strain are opsonic to both CPS-A and CPS-B strains. LTA-specific antibodies also bind to LTA of CPS-C and CPS-D strains, but fail to opsonize them. From CPS-C and CPS-D strains resistant to opsonization by anti-LTA, we purified a novel diheteroglycan with a repeating unit of →6)-β-Gal*f*-(1→3)- β-D-Glc*p*-(1→ with *O*-acetylation in position 5 and lactic acid substitution at position 3 of the Gal*f* residue. The purified diheteroglycan, but not LTA absorbed opsonic antibodies from whole cell antiserum against *E. faecalis* type 2 (a CPS-C strain) and type 5 (CPS-D). Rabbit antiserum raised against purified diheteroglycan opsonized CPS-C and CPS-D strains and passive protection with diheteroglycan-specific antiserum reduced bacterial counts by 1.4 – 3.4 logs in mice infected with *E. faecalis* strains of the CPS-C and CPS-D serotype. Diheteroglycan-specific opsonic antibodies were absorbed by whole bacterial cells of *E. faecalis* FA2-2 (CPS-C) but not by its isogenic acapsular *cpsI-*mutant and on native PAGE purified diheteroglycan co-migrated with the gene product of the *cps*-locus, suggesting that it is synthesized by this locus. In summary, two polysaccharide antigens, LTA and a novel diheteroglycan, are targets of opsonic antibodies against typeable *E. faecalis* strains. These cell-wall associated polymers are promising candidates for active and passive vaccination and add to our armamentarium to fight this important nosocomial pathogen.

## Introduction

The classification system for streptococci was developed by Rebecca Lancefield at the beginning of the 20th century [1]. Enterococci were assigned to the serogroup D in the Lancefield typing system, and the group-specific antigen was subsequently identified as lipoteichoic acid (LTA) by Wicken and colleagues [2]. About fifty years later, our group demonstrated that antibodies that opsonize *E. faecalis* strain 12030 bind to the group antigen LTA [3]. In a recent serotyping system based on carbohydrate-specific antibodies, 60% of *E. faecalis* strains were typeable and assigned to four serotypes, designated CPS-A to CPS-D [4]. However, the structural equivalents of the type-specific antigens in this serotyping system are still unknown. This is surprising because several major carbohydrate structures of the enterococcal cell wall were described by Pazur, Bleiweis, and Krause in a number of landmark studies almost forty years ago [5], [6], [7], [8]. These authors identified two major glycans from cell wall extracts of *E. faecalis*: a rhamnopolysaccharide (also called tetraheteroglycan or cell wall polysaccharide) and a diheteroglycan. The rhamnopolysaccharide was first described by Elliott et al. as the type antigen of *Streptococcus faecalis* in 1960 [9]. Bleiweis and Krause characterized the type antigen in more detail and reported that it is a complex carbohydrate containing rhamnose, glucose, glucosamine, and galactosamine as well as ribitol and phosphorus [8]. Rhamnopolysaccharides of similar composition were also described by Pazur and Karakawa [6] and in two more recent studies [10], [11]. In the early 1970ies, Pazur et al also isolated a polysaccharide containing glucose and galactose from *E. faecalis* and named it diheterglycan [5], [6]. A number of more recent studies have investigated genetic loci involved in the biosynthesis of polysaccharides of the enterococcal cell envelope, but chemical structures of the respective polysaccharides and their potential as vaccine antigens have not been explored [11], [12], [13], [14], [15], [16], [17], [18].

Enterococcus is currently ranked third among Gram-positive pathogens to cause hospital-associated infections in the US [19] and was the second most common pathogen isolated from ICU patients worldwide in a point-prevalence study conducted in 2007, causing 10% of nosocomial infections on the ICU [20]. With limited options in antimicrobial chemotherapy available, a renewed interest in alternative treatment and prevention strategies such as active and passive immunization has evolved. Capsular polysaccharides have been highly successful vaccine antigens for vaccines against various bacterial pathogens, but little is known about cell envelope polysaccharides as a target of protective antibodies against *E. faecalis*. Hancock, Gilmore and Thurlow [12], [13], [14], [15] described a capsule in *E. faecalis* synthesized by the *cps* locus. This capsule mediates resistance to killing by serum and neutrophils or macrophages, augments bacterial persistence in vivo, and impedes C3b deposition on the bacterial surface [4], [14]. However, to date, no definite chemical structure of the capsular polysaccharide has been published.

In the current study, we have revisited the cell wall carbohydrates of *E. faecalis* and investigated their role as antigens in the CPS-serotyping system by Hufnagel and colleagues. Using highly purified polysaccharides, we were able to show that opsonic antibodies are directed against only two of these antigens: In acapsular strains, LTA is the major opsonic epitope and in encapsulated strains opsonic antibodies bind to a novel diheteroglycan, the putative capsular polysaccharide of *E. faecalis* in CPS-C and CPS-D strains.

## Results

### CPS-A and CPS-B strains but not CPS-C and CPS-D strains are opsonized by LTA-specific antibodies

**Figure 1:**
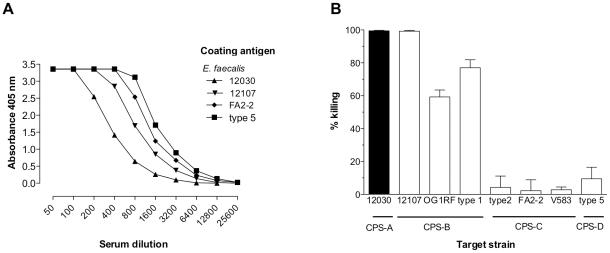
Cross-reactivity of antibodies directed against enterococcal LTA. (**A**) Binding of rabbit IgG raised against LTA purified from *E. faecalis* strain 12030 to LTA extracted from *E. faecalis* strains of various CPS-serotypes. The coating antigens and serum dilutions are specified in the legend. Each point represents the average of two determinations. (**B**) Opsonophagocytic killing of various *E. faecalis* strains by the same anti-LTA rabbit antiserum. A serum dilution of 1∶800 was used to assess killing activity. The respective target strains are indicated in the legend. Opsonophagocytic killing activity was compared to controls from which leukocytes were omitted. Each bar represents the mean of four determinations and the error bar the SEM.

We reported previously that *E. faecalis* strains belonging to the CPS-A serotype are opsonized by antibodies specific to a teichoic-acid like polysaccharide, which was later shown to be structurally identical to LTA [3], [4]. More than half of the *E. faecalis* strains, however, belong to serotypes CPS B – D and are not opsonized by this antiserum [4], [21]. To further explore the role of antibodies against LTA in the serodiversity of *E. faecalis* strains, we vaccinated a rabbit with LTA from *E. faecalis* 12030 (CPS-A), which was extracted and purified using non-degrading conditions. In a western blot analysis with whole cell lysates of the vaccine strain this antiserum was monospecific to LTA (data not shown). In the opsonophagocytic killing assay, anti-LTA antibodies mediated killing not only of CPS-A strains, but also of *E. faecalis* strains that belong to the CPS-B serotype. In contrast, CPS-C and CPS-D strains were not opsonized (Fig. 1B). Next, we wanted to explore if CPS-C and CPS-D strains may express a structurally distinct LTA molecule that is not recognized by antibodies that are raised against LTA from a CPS-A strain. We purified LTA from three *E. faecalis* strains that belonged to the serotypes CPS-B – CPS-D and measured binding by ELISA (Fig. 1A). Anti-LTA bound equally well, however, to LTA derived from CPS-A (*E. faecalis* 12030), CPS-B (12107), CPS-C (FA2-2) and CPS-D (type 5) strains, suggesting that antigenic variability of LTA is irrelevant for the lack opsonic activity of LTA antiserum against CPS-C and CPS-D strains (Fig. 1, for specifications of *E. faecalis* strains see table 1).

**Table 1:** *E. faecalis* strains used in the study.

Strain	Serotype	Source	MLST*	Synonym	Reference
*E. faecalis* 12030	CPS-A	clinical	64		[22]
*E. faecalis* 12107	CPS-B	clinical	21		[22]
*E. faecalis* OG1RF	CPS-B	oral	1		[45]
*E. faecalis* type 1	CPS-B	unknown	21	MCTC 8727	[26]
*E. faecalis* type 2	CPS-C	urine	11	MCTC 8796	[26]
*E. faecalis* type 21	CPS-C	infant/fecal	30	MCTC 8746	[26]
*E. faecalis* R19.001	CPS-C	fecal	unknown		[46]
*E. faecalis* V583	CPS-C	blood	6	ATCC700802	[47]
*E. faecalis* FA2-2	CPS-C	clinical	8		[48]
*E. faecalis* HG101	–	*cpsI* mutant of FA2-2	–		[12]
*E. faecalis* type 5	CPS-D	urine	68	MCTC 8731	[26]

*for reference, see [21].

### Purification of a novel capsular polysaccharide in CPS-C and CPS-D strains

Since CPS-C and CPD-D strains are not killed by anti-LTA antibodies, we hypothesized that LTA in these strains is masked by a polysaccharide capsule, an assumption also supported by agglutination experiments by Thurlow and coworkers [15]. To investigate this hypothesis, we released cell wall associated carbohydrates in CPS-C (*E. faecalis* FA2-2 and *E. faecalis* type 2) and CPS-D (*E. faecalis* type 5) strains by enzymatic digestion of peptidoglycan and separated the extracted material by gel-permeation chromatography. Carbohydrate eluting at void volume consisted of LTA as determined by ^1^H NMR analysis. A large second peak eluting around a K_av_ of 0.45 was further purified by anion-exchange chromatography. The majority of the material eluted from Q-Sepharose around 300 mM NaCl and contained rhamnose, glucose, galactose, *N*-acetylglucosamine and *N*-acetylgalactosamine, as determined by sugar analysis. The compositional analysis of this polysaccharide was consistent with the previously described rhamnopolysaccharide [10], [11], [12], [16], [22]. A smaller, adjacent peak eluted at 450 mM NaCl and contained only glucose and galactose. This material migrated as a single, broad band around 100 kDa on SDS-PAGE electrophoresis and stained positive with periodic acid-Schiff (PAS) but not with Commassie blue (Fig. S1). Preliminary analysis by ^1^H-NMR spectroscopy revealed that this material contained a novel diheteroglycan and impurities of lipoteichoic acid, which were removed by a final purification step using gel-permeation chromatography on a Toyopearl column HW-40S. This final preparation contained <3% protein and <1% phosphorus and was used for consecutive chemical and biological analysis.

### Structural analysis of capsular polysaccharide from *E. faecalis* CPS-C and CPS-D strains reveals a novel diheteroglycan

**Figure 2:**
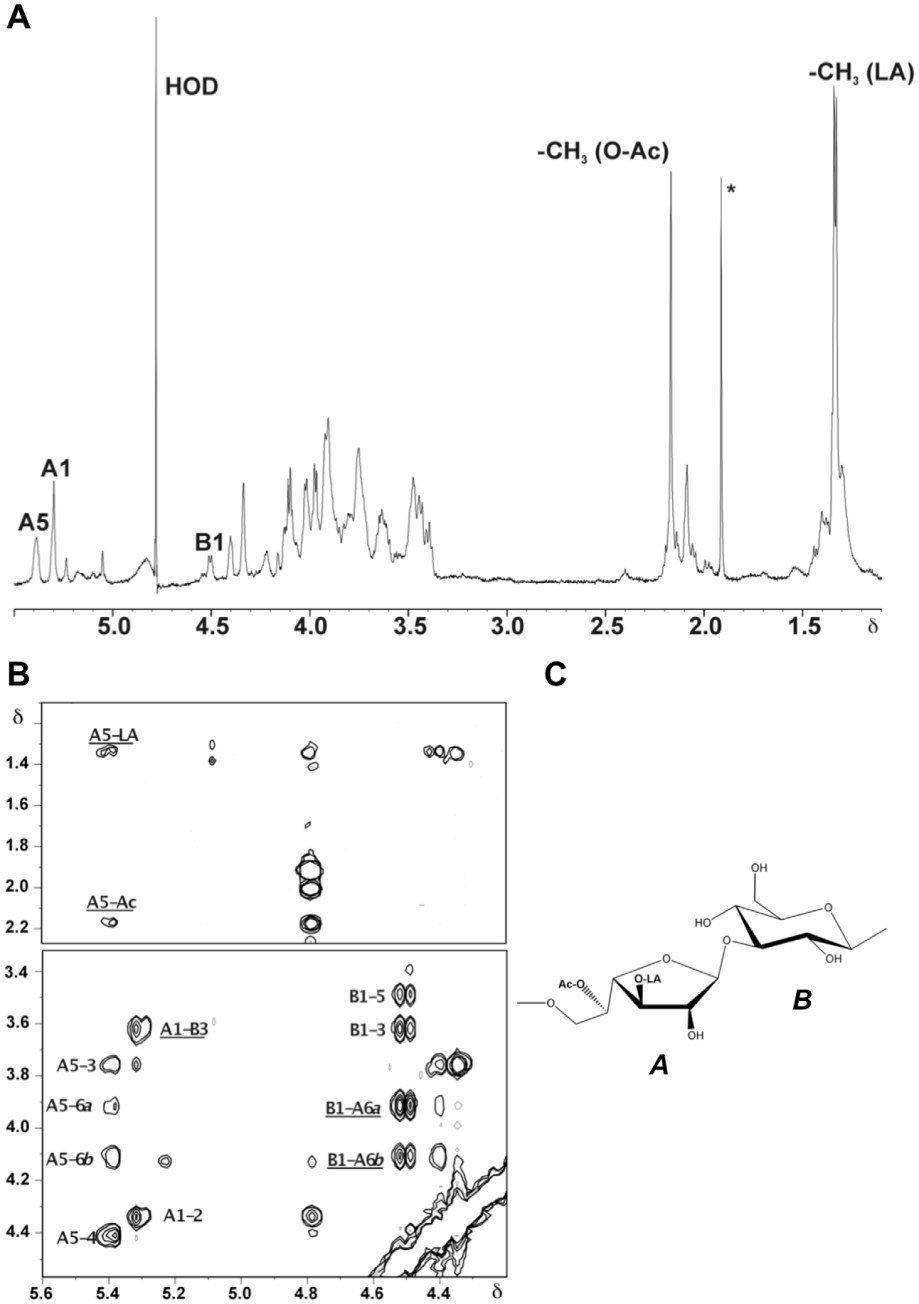
Structural characterization of diheterglycan by nuclear magnetic resonance (NMR) analysis. (**A**) The ^1^H NMR spectrum of diheteroglycan isolated from *E. faecalis* type 2. The spectrum was recorded at 600 MHz and 27°C. The *letters* refer to the carbohydrate residues as shown in chemical structure (Fig. 2C), and the *numbers* refer to the protons in the respective residues; LA, lactic acid. (**B**) Sections of the ROESY spectrum of *E. faecalis* diheteroglycan. The *inter*residual NOE contacts are underlined. (**C**) Chemical structure of the repeating unit of *E. faecalis* capsular diheteroglycan. * Acetic acid remain of the final gel-permeation chromatography step.

Compositional analysis identified the presence of D-Glc. The ^1^H NMR spectrum of the diheteroglycan isolated from *E. faecalis* type 2 (CPS-C, Fig. 2A) showed two anomeric signals at δ 5.297 (residue A, {^3^ *J*_H1,H2_ <2 Hz}), and at δ 4.491 (residue B, {^3^ *J*_H1,H2_ = 7.8 Hz}), which were assigned to H-1 of β-Gal*f* and β-Glc*p*, respectively. In addition, a broad signal at δ 5.379 was identified, which was assigned to proton H-5 of β-Gal*f* due to the substitution of position C-5 by an *O*-acetyl group (δ 2.164). Furthermore, the doublet at δ 1.366 was recognized as methyl group belonging to lactic acid (LA) residue [23], [24], [25].

All ^1^H and ^13^C chemical shifts of the capsular polysaccharides were established from ^1^H,^1^H correlation and total correlation as well as ^1^H,^13^C heteronuclear multiple quantum coherence NMR experiments. Low-field shifted signals of carbon atoms demonstrated substitutions at C-6 and C-3 of β-Gal*f* (δ 69.63 and δ 84.85, respectively) and substitution at C-3 of β-Glc*p* (δ 82.58) The chemical shifts are summarized in table S1.

The sequence of the residues in the repeating unit of the capsular polysaccharides was established by rotating frame nuclear Overhauser effect (NOE) spectroscopy (ROESY, Fig. 2B) and heteronuclear multiple bond correlation (HMBC, Fig. S2) experiments. Strong *inter*residual NOE contacts were observed between H-1 **A/**H-3 **B** (δ 5.297/3.622), as well as between H-1 **B**/H-6a **A** (δ 4.491/3.897), and H-1 **B**/H-6b **A** (δ 4.491/4.101). Additional weak NOE contacts were found between H-5 **A** and **LA** methyl group (δ 5.379/1.336), and H-5 **A** and *O*-acetyl methyl group (δ 5.379/2.164). The HMBC data confirmed the sequence of the constituents assigned from ROESY data. The following *inter*residual proton-carbon correlations were observed: H-1 **A**/C-3 **B** (δ 5.297/82.58), C-1 **A**/H-3 **B** (δ 109.20/3.622), C-1 **B**/H-6a **A** (δ 103.44/3.897), as well as H-3 **A**/C-2 **LA** (δ 3.747/77.88), and C-3 **A**/H-2 **LA** (δ 84.85/3.966). The chemical structure of the isolated polysaccharide is shown on the Fig. 2C.

^1^H NMR spectroscopy of diheteroglycans isolated from *E. faecalis* FA2-2 (CPS-C) and type 5 (CPS-D) revealed a polysaccharide that they only differed from the *E. faecalis* type 2 (CPS-C) diheteroglycan by their lack of *O*-acetylation of β-Gal*f* (for structural analysis of diheteroglycan from type 5 see Fig. S3, S4 and table S2). In subsequent experiments we isolated *O*-acetylated and *O*-deacetylated diheteroglycan from *E. faecalis* type 2 using the same culture conditions. Thus, it cannot be excluded that the chosen purification scheme may result in the loss of the labile *O*-acetyl group during the isolation of the polysaccharide.

### Antibodies against diheteroglycan are opsonic to CPS-C and CPS-D strains

**Figure 3:**
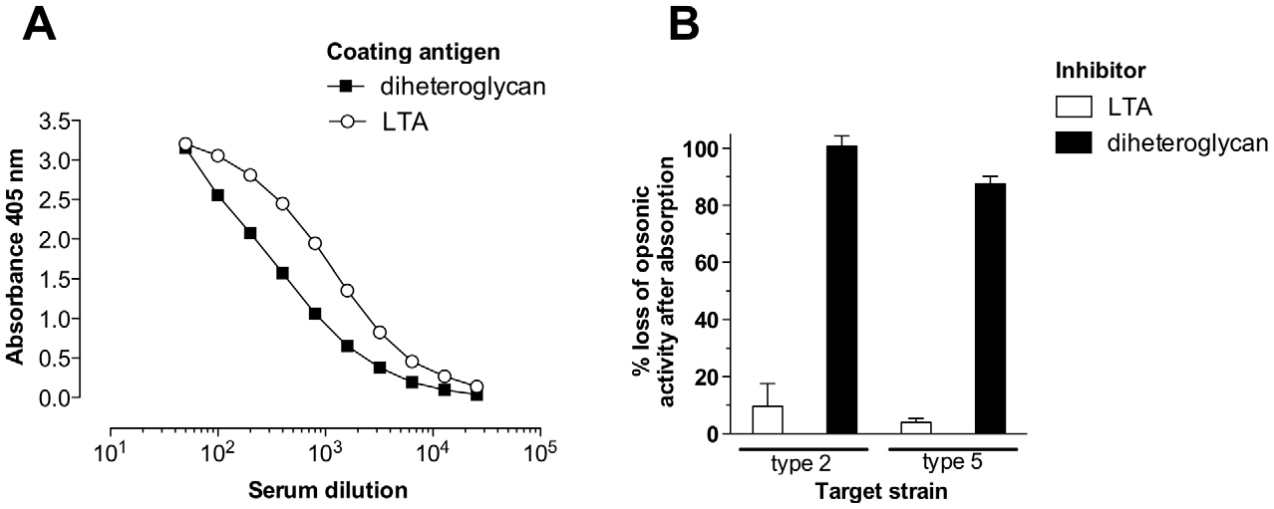
Specificity of antibodies raised by vaccination with whole bacterial cells of *E. faecalis*. (**A**) Binding of rabbit IgG raised against whole bacterial cells of *E. faecalis* type 2 to diheteroglycan and LTA purified from the same strain. The coating antigen and serum dilutions are indicated in the legend. (**B**) Absorption of opsonic activity against *E. faecalis* type 2 (CPS-C) and type 5 (CPS-D) by cell envelope carbohydrate antigens. Rabbit antiserum raised against whole bacterial cells of the respective target strain was used. For the inhibition opsonophagocytic killing assay, rabbit antiserum was preincubated for 60 min with 10 µg/ml of either LTA or diheteroglycan purified from the homologous *E. faecalis* strain and used at a final dilution of 1∶800 in the assay. Bars are means and error bars the SEM four determinations.

**Figure 4:**
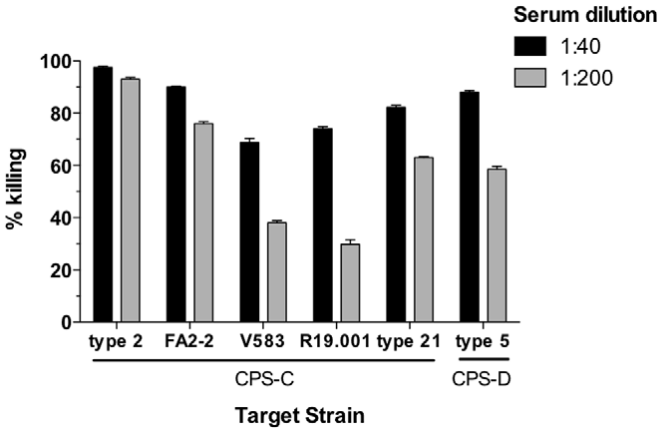
Opsonophagocytic killing of *E. faecalis* CPS-serotype C and D strains by rabbit antiserum after immunization with diheteroglycan purified from *E. faecalis* type 2 (CPS-C). Serum dilutions were used as indicated in the legend. Bars represent the mean of four determinations and the error bar the SEM.

We previously generated antisera by vaccination with heat-killed, proteinase K digested bacterial cells of *E. faecalis* type 2 (CPS-C) and type 5 (CPS-D) that opsonize CPS-C or CPS-D strains [4]. The antisera contained antibodies that bound to LTA but also antibodies that recognized enterococcal diheteroglycan (Fig. 3A). Using both antigens as inhibitors in the opsonophagocytic killing assay, we assessed the specificity of opsonic antibodies of the antisera against *E. faecalis* type 2 and type 5 (Fig. 3B). In agreement with results obtained with antiserum against LTA showing no opsonic activity against type 2 and type 5 strains, purified LTA was also a poor inhibitor (i.e. <10% inhibition) of opsonophagocytic killing mediated by the serotype-specific antiserum. Purified diheteroglycan, on the other hand, inhibited 98% of opsonic activity of antiserum against *E. faecalis* type 2 (CPS-C) and 88% of opsonic killing of *E. faecalis* type 5 (CPS-D) by the respective antiserum indicating that the majority of opsonic antibodies raised by whole cell vaccination are directed against this antigen. To further explore the potential of diheteroglycan as candidate for an enterococcal vaccine, we immunized a rabbit with purified antigen from *E. faecalis* type 2 (CPS-C). Western blot analysis of this antiserum with whole cells lysates of the same strain as antigen confirmed the presence of antibodies against the high molecular weight band of diheteroglycan. In addition, we detected antibodies against a second, broad band that migrated around 20–30 kDa, suggestive of antibodies against LTA (data not shown). Diheteroglycan – like many other bacterial polysaccharide antigens – was overall poorly immunogenic and induced only moderate levels of specific antibodies as quantified by ELISA (Fig. S5). We further investigated cross-reactivity of diheteroglycan-specific antibodies by the opsonophagocytic killing assay (Fig. 4). At a serum dilution of 1∶40, diheteroglycan-specific antibodies were opsonic to all *E. faecalis* strains of the CPS-C and CPS-D serotype evaluated (i.e. killing >70%). At higher serum dilutions, opsonic killing activity of ≥50% was observed only for three out of five heterologous *E faecalis* strains, indicating overall moderate titers of opsonic antibodies induced by vaccination with diheteroglycan (Fig. 4).

### Diheteroglycan shares antigenic and structural similarities with the gene product of the *cps* locus

**Figure 5:**
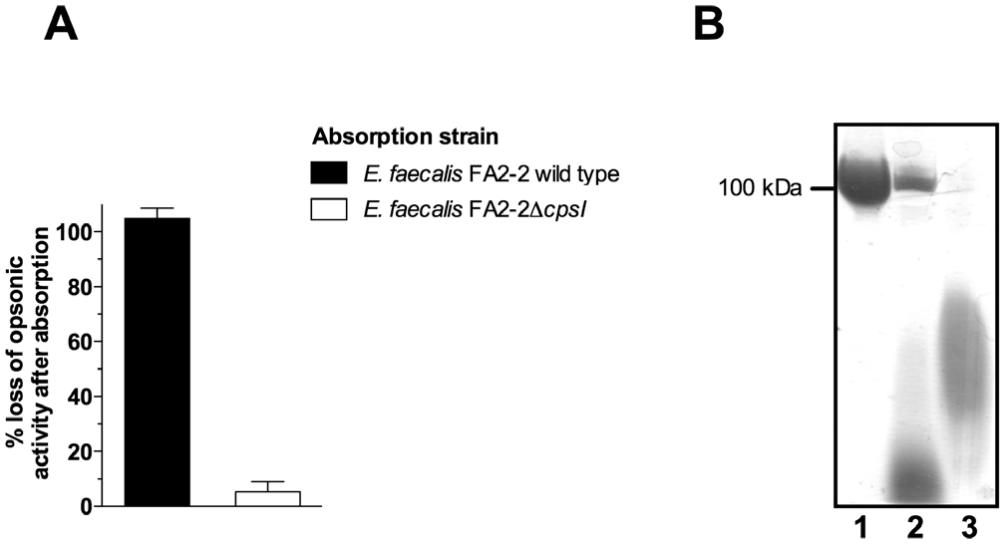
Relationship of diheteroglycan to the biosynthetic product of the *cps* locus. (**A**) Loss of opsonic activity after absorption of rabbit antiserum raised against *E. faecalis* type 2 (CPS-C) diheteroglycan with whole bacteria. Before the assay the serum was absorbed with either *E. faecalis* FA2-2 (CPS-C) wild type or its isogenic, acapsular *cpsI* mutant (*E. faecalis* HG101) for 60 min. Absorbed serum was used at a final dilution of 1∶40 in the assay. Bars represent the mean of four determinations and the error bar the SEM. (**B**) Native PAGE of purified diheteroglycan from *E. faecalis* type 2 (lane 1), cell wall lysates of *E. faecalis* FA2-2 wild type (CPS-C, lane 2) and the isogenic *cpsI* mutant (HG101, lane 3). Cell envelope carbohydrates were released by digestion of peptidoglycan by lysozyme and mutanolysin and acrylamide gels were stained with Stains-All according to the method of Hancock et al. [12].

Hancock and Gilmore previously described a gene locus involved in the biosynthesis of a putative capsular polysaccharide of *E. faecalis* strains of the CPS-C and CPS-D serotype [12], [14], [15]. The product of the *cps*-locus is a capsular polysaccharide composed of glucose, galactose, glycerol, and phosphate [12], [15]. To examine antigenic similarity of diheteroglycan and the capsular polysaccharide produced by the *cps* locus, we absorbed the antiserum raised against diheterglycan derived from *E. faecalis* type 2 (CPS-C) with an acapsular *cpsI* mutant in *E. faecalis* FA2-2 and the isogenic wild type strain (CPS-C) [12]. Absorption of diheteroglycan-specific rabbit antiserum with the wild-type *E. faecalis* but not with the acapsular mutant abolished opsonic killing of the *E. faecalis* FA2-2 (Fig. 5A). For comparison of the biosynthetic product of the *cps*-locus with diheteroglycan we released cell wall carbohydrates from *E. faecalis* FA2-2 and its isogenic *cpsI* mutant and applied it along with purified polysaccharide to acrylamide gel electrophoresis (Fig. 5B). Electrophoretic mobility of diheteroglycan was identical to a high molecular weight band around 100 kDa that was present in *E. faecalis* FA2-2 wild type strain but not in the *cpsI* mutant (Fig. 5B).

### Antibodies to diheteroglycan protect against bacteremia in mice

**Figure 6:**
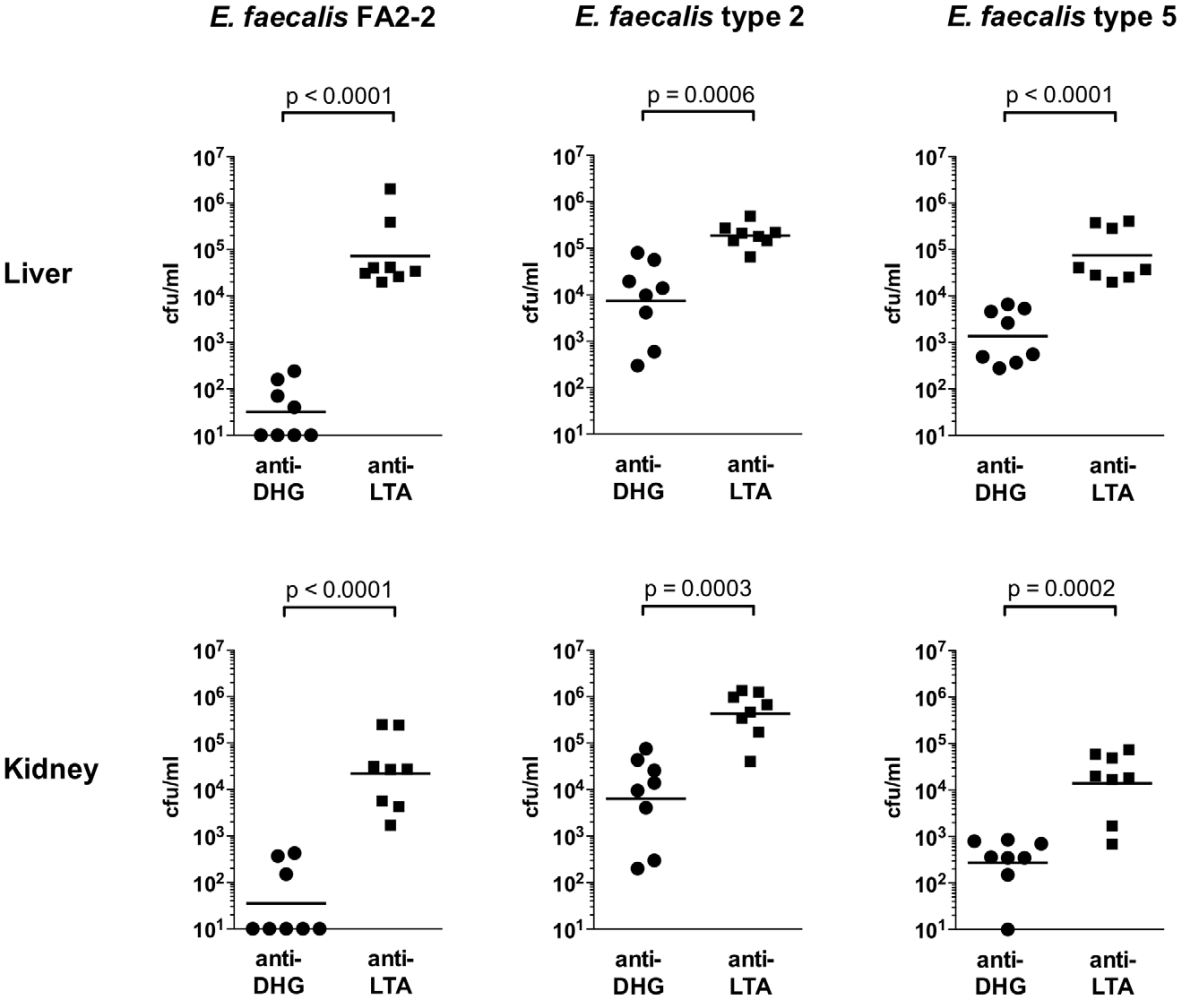
Passive protection by rabbit antiserum in a mouse bacteremia model. Six to eight weeks old female Balb-C mice were passively immunized by i.p. injection of 200 µl of heat-inactivated rabbit antiserum raised against diheterglycan (anti-DHG) from *E. faecalis* type 2 or LTA purified from *E. faecalis* 12030 (anti-LTA) 24 and 12 h before and 4 h after infection. Fourty-eight h after i.v. injection of bacteria via the tail vein, mice were sacrificed and bacterial counts quantified. Mice were infected with *E. faecalis* FA2-2 (3.0×10^9^ cfu per mouse), *E. faecalis* type 2 (2.0×10^9^) and *E. faecalis* type 5 (2.5×10^9^) as indicated in the graph. Bars represent geometric means. Seven to eight mice per group were used. The lower limit of detection was 10 CFU/ml. Groups of mice were compared using the T-test of log-transformed CFU counts.

Having demonstrated that diheteroglycan-specific antibodies are opsonic to *E. faecalis* CPS-C and CPS-D strains, we assessed in vivo protection by passive immunization in a mouse bacteremia model. To this end, BALB-C mice received rabbit antibodies against diheteroglycan or against LTA (as a control) 48 h and 24 h before and 4 h after i.v. challenge. A total of three *E. faecalis* strains were evaluated (*E. faecalis* type 2 and FA2-2 (CPS-C), type 5 [CPS-D]). Anti-LTA was chosen as control because natural LTA-specific antibodies were present in the diheteroglycan antiserum and because identical vaccination protocols including complete Freunds adjuvant were used for production of both antisera. Forty-eight hours after infection, bacterial counts from the blood, kidneys and liver were enumerated. At this time point, mice of both immunization groups had cleared the bacteremia, but – depending on the *E. faecalis* challenge strain - bacterial counts in the kidney and liver were reduced 1.4 to 3.4 logs in mice immunized with diheteroglycan-specific antibodies compared to mice that had received rabbit anti-LTA (Fig. 6).

## Discussion

Defining serotypes and corresponding structures of the cell envelope that constitute the basis of serospecificity is a critical step in vaccine development. Using formaldehyde-killed bacteria for immunization, in 1992, Maekawa identified 21 serotypes of *E. faecalis* by cross-agglutination and absorption studies [26]. More recently, Hufnagel defined a simplified serotyping system based largely on carbohydrate antigens of *E. faecalis* [4]. Prototype sera for Hufnagel's study were generated by vaccination with purified polysaccharide or heat-killed, proteinase K-digested whole bacteria, and strains were typed according to cross-reactivity measured by ELISA and the opsonophagocytic killing assay. Using this methodology, 60% of a total of 29 clinical *E. faecalis* isolates could be assigned to one of four serotypes, CPS-A to CPS-D [4]. However, serospecificity of opsonic antibodies was not unequivocal between CPS-A and CPS-B strains or between CPS-C and CPS-D strains [4]. McBride and Gilmore examined the diversity of capsule expression in *E. faecalis* on a genetic basis in 106 strains of diverse origin and found that approximately half of the strains lacked genes of the *cps* locus that are essential for capsule production [21].

Our group has demonstrated previously that the teichoic acid-like cell envelope polysaccharide that is the target of opsonic antibodies in CPS-A strains is structurally identical to LTA [3]. Using the highly purified LTA obtained from our previous study we reassessed the cross-reactivity of LTA-specific antibodies. In a previous publication by us, we demonstrated that opsonic antibodies against CPS-A and CPS-B serotypes are not cross-reative [4]. In our current study antibodies raised against purified LTA reacted with both CPS-A strains (*E. faecalis* 12030) and CPS-B strains (*E. faecalis* 12107, OG1RF, type 1). The discrepancy of our current and previous study may be related to the different antigen preparations used for production of rabbit antisera. For a previous investigation we utilized a partially deacylated and dealanylated LTA molecule that may have lost antigenic properties during the purification process [3], [4]. Our finding that CPS-A and CPS-B only represent a single serotype is supported by genetic characterization of the *cps*-locus: CPS-A and CPS-B strains both contain only the first two *cps*-genes (*cpsA* and *cpsB*) but lack remaining the genes of this locus (*cpsC* – *cpsK*) which are essential for capsule production [4], [15]. Also, LTA-specific antibodies agglutinate CPS-A and CPS-B but not CPS-C and CPS-D strains, indicating that LTA is surface exposed in the former serotypes [15].

In contrast to acapsular CPS-A and CPS-B strains, our and Thurlow's data indicate that LTA of CPS-C and CPS-D strains is not available on the bacterial surface to bind specific antibodies for opsonization via the classical pathway, probably because a polysaccharide capsule masks this antigen [14]. With evidence suggesting the presence of an antiphagocytic capsule in *E. faecalis*, we next purified cell envelope polysaccharides from the CPS-C strain *E. faecalis* type 2 and identified a novel diheteroglycan.

The isolated diheteroglyan represented only a small proportion of carbohydrates released by enzymatic digestion of peptidoglycan. Most of the material obtained by this mode of extraction was a rhamnopolysaccharide with similar composition as described previously [8], [9], [10], [11], [12], [22]. The diheterglycan described here eluted in close association with the rhamnopolysaccharide and LTA during the chromatographic separation process, making it challenging to isolate this carbohydrate in high purity. Capsule extracts from two additional *E. faecalis* strains (FA2-2 [CPS-C] and type 5 [CPS-D]) contained a polysaccharide with an identical repeating unit of →6)- β-Gal*f*-(1→3)- β-D-Glc*p*-(1→. In *E. faecalis* type 2 we recovered this polysaccharide with and without *O*-acetylation in position C-5 of Gal*f*, while the polysaccharide isolated from *E. faecalis* type 5 and FA2-2 lacked *O*-acetylation. Since *O*-acetyl substituents are pH labile and hydrolyzed under mild basic conditions, the *O*-deacetylated diheteroglycan may represent an artifact of the conditions we have chosen for column chromatography. Alternatively, we cannot exclude that the degree of *O*-acetylation varies due to minor differences in culture conditions between the batches used for purification. O-acetyl groups may influence the biochemical properties of carbohydrates (e.g. solubility in water). They also can be part of antigenic epitopes of bacterial polysaccharides recognized by opsonic antibodies [27] but may also mask them [28].

Pazur et al. described a diheteroglycan of very similar composition in *E. faecalis* [5], [6]. The proposed structure of Pazur's diheteroglycan is a backbone consisting of a trisaccharide repeating unit of →4)-β-Glc-(1→6)-β-Glc-(1→4)-β-Gal-(1→ substituted with lactosyl and cellobiosyl side chains attached by β-(1→4) linkages to alternate glucose residues of the backbone [5]. Since this structure was determined before the availability of NMR spectrometry for more definitive carbohydrate analysis, it is tempting to speculate that this molecule is in fact identical with the carbohydrate identified by us. The composition of our diheteroglycan also bears resemblance to the capsular polysaccharide described by Hancock and Gilmore [12], [15] although these authors never reported complete structural information for their antigen. Their capsular polysaccharide is synthesized by the *cps* locus and was identified as a 130 kDa glycan. The composition of the material isolated from strain FA2-2 (CPS-C) was determined to be glucose, galactose, glycerol and phosphate at a ratio of 4∶1∶1∶2 [12]. Serum absorptions experiments, acrylamide gel elecrophoresis and structural data presented here suggest that both polysaccharide antigens – diheteroglycan and capsular polysaccharide produced by the *cps* locus - are identical. Our studies demonstrate that opsonic antibodies directed against diheteroglycan bind to capsule-bearing CPS-C strains, but not to its isogenic acapsular mutant. Also, both antigens co-migrate in native PAGE and contain glucose and galactose, albeit at different ratios. We can only speculate about the reason for this discrepancy in monosaccharide composition, but impurities in the capsular polysaccharide described by Hancock may explain the compositional differences. As mentioned above, the diheteroglycan partially co-elutes during the chromatography process with LTA and only an additional purification step following ion exchange chromatography yielded pure material in our study. Hence, impurities of LTA could explain why Hancock and Gilmore found more glucose, glycerol and phosphate in their preparations of capsular polysaccharide. Of note, high-pH anion-exchange chromatography–pulsed-amperometric detection employed for compositional analysis in Hancock's study is not suitable to detect LTA in mixtures of complex carbohydrates [12], [15].

To test if capsule-specific antibodies can protect against *E. faecalis* infection, we passively immunized mice with either anti-diheteroglycan or anti-LTA rabbit serum and challenged them with *E. faecalis* CPS-C and CPS-D strains intravenously. Bacterial counts were 1.4–3.4 logs lower in kidneys and livers of mice immunized with anti-diheteroglycan for the three strains tested. The variable protective efficacy against the tested *E. faecalis* strains is somewhat surprising, but may be explained by differences of in vivo expression or the degree of O-acetylation of diheteroglycan between strains. In broader terms, this level of reduction of bacterial load by passive protection is comparable if not superior to vaccine efficacy achieved for various vaccine antigens evaluated in *Staphylococcus aureus* [29], [30], [31], [32]. It is difficult to predict, if this level of vaccine efficacy in mice will translate into protection in the human host. In contrast to other Gram-positive pathogens like *Streptococcus pneumoniae* or group-B streptococci, which are virulent pathogens in humans and mice, mice clear enterococcal bacteremia or peritonitis spontaneously unless a very high inoculum (>10^8^–10^9^ bacteria per mouse) is given. Hence, in our animal model we may have overwhelmed adaptive immunity with bacterial loads much higher than encountered during human infection, a problem that also riddles vaccine research for other nosocomial pathogens of low virulence (e.g. *Staphylococcus epidermidis*, *Candida spp.*). Nevertheless, we expect that conjugation of the diheteroglycan capsule to a protein carrier will enhance immunogenicity of the vaccine and elicit better protection in vivo. Active and passive vaccination strategies with a diheteroglycan conjugate vaccine could potentially be employed in patients that are at high risk of invasive enterococcal infections such as patients hematologic malignancies, neutropenia, or liver-transplant recipients [33], [34], [35].

In summary, the results presented in this study provide evidence that two carbohydrates of the cell envelope are targets of opsonic antibodies in typeable strain of *E. faecalis*. Active vaccination with LTA and diheteroglycan or a passive immunotherapy approach using recombinant human monoclonal antibodies could therefore target non-encapsulated and capsule-expressing strains and may provide protection against a majority of *E. faecalis* strains.

## Materials and Methods

### Bacterial strains and culture

Bacterial strains and the respective serotype according to Hufnagel et al. are specified in table 1. Unless otherwise indicated, bacterial cells were grown from starter cultures in Columbia broth (Becton Dickinson, Sparks, MD, USA) supplemented with 1% glucose at 37°C until they reached an optical density at 600 nm of 0.6 to 0.8 and harvested by centrifugation.

### Antisera

Rabbit antisera against whole bacterial cells of *E. faecalis* type 2 and type 5 have been described previously [4]. Anti-LTA rabbit antiserum was prepared using LTA purified from *E. faecalis* 12030 by butanol extraction and hydrophobic interaction chromatography [3]. Antiserum against diheteroglycan and LTA was raised by s.c. immunization of one female New Zealand white rabbit for each antigen with 100 µg of antigen suspended in complete Freund's adjuvant followed by the same dose in incomplete Freund's adjuvant seven days later and intravenous booster doses of 10 µg every three days in the consecutive week. Antiserum was obtained five weeks after the beginning of the immunization.

### Preparation and characterization of capsular polysaccharide

Diheteroglycan of *E. faecalis* was isolated by methods similar to those described previously [3], [22]. Briefly, *E. faecalis* strains type 2, FA2-2 and type 5 were cultivated as described above and harvested by centrifugation. The bacteria were washed in PBS and cell walls were digested by addition of mutanolysin and lysozyme (each at 100 µg/ml, Sigma Chemicals, St. Louis, MO, USA in PBS supplemented with 5 mM MgCl_2_, 1 mM CaCl_2_ and 0.05% NaN_3_) at 37°C for 18 h. Insoluble material was removed by centrifugation, and the supernatant was treated with nucleases (DNase I and RNase A, 100 µg/ml) at 37°C for 4 h followed by addition of proteinase K (100 µg/ml, all Sigma Chemicals) at 56°C for 18 h. The supernatant was precipitated by the addition of ethanol (80% final volume), collected by centrifugation, dialyzed against deionized H_2_O, and lyophilized. For size exclusion chromatography, the material was redissolved in 0.01 M ammonium carbonate buffer (pH 8.0) and applied to a column (1.6×90 cm) of Sephacryl S-400 (GE Healthcare, Uppsala, Sweden). Fractions eluting at around a K_av_ of 0.45 were combined, dialyzed and lyophilized. The material was resuspended in 20 mM NaHCO_3_, pH 8.4 and applied to an anion-exchange column (Sepharose Q FF, GE Healthcare). Bound antigen was eluted from the column by a linear gradient of 0–1 M NaCl and fractions were assayed for hexose content by the Dubois assay and for phosphorus using the Lowry method [36], [37]. Hexose-positive and phosphorus-negative fractions eluting at 450 mM NaCl were combined, dialyzed and lyophilized. As a final purification step, gel-permeation chromatography was performed on a 1.5×75 cm Toyopearl HW-40 column (Tosoh Corporation, Tokyo, Japan).

### Preparation of LTA

LTA was prepared by butanol extraction and hydrophobic interaction chromatography as described previously [3]. LTA preparations were evaluated for purity by the Bradford assay, SDS-PAGE and western blot analysis with the respective antiserum to whole bacterial cells (see above). Structural identity of LTA was confirmed by NMR spectroscopy as described recently [3].

### Chemical characterization of *E. faecalis* diheteroglycan

Protein and phosphorus content of purified diheteroglycan as quantified using standard assays [36], [38]. The polysaccharide was further characterized by SDS-PAGE in gradient gels (4/12% w/v, Invitrogen), followed by staining for proteins with Coomassie blue and with the PAS reaction for carbohydrates [39]. Compositional analyses were performed as described previously [3], [40], [41].

### Polyacrylamide gel electrophoresis

Cell wall carbohydrates were released from *E. faecalis* FA2-2 or HG101 by treatment with mutanolysin, lysozyme and nuclease and proteinase K treatment as described above. Next, 25% ethanol was added and precipitated material was discarded. More ethanol was added to a final concentration of 75% for the precipitation of carbohydrates. Purified diheteroglycan and cell wall extracts were analyzed by electrophoresis through 3% polyacrylamide (33∶1) in Tris-borate buffer (0.2 M Tris-base/0.2 M boric acid/20 mM EDTA, pH 8.3), and detected using Stains-All (3,3′-dimethyl-9-methyl-4,5,4′5′-dibenzothiacarbocyanine) according to the method of Hancock et al. [12].

### NMR spectroscopy

Samples were exchanged three times with 99.90% ^2^H_2_O, lyophilized, and redissolved in 99.99% ^2^H_2_O. All one-dimensional and two-dimensional spectra were recorded at 27°C with a Bruker DRX Avance 600 MHz spectrometer as described previously [3]. Chemical shifts were reported relative to internal acetone (δ_H_ 2.225; δ_C_ 31.45).

### ELISA studies

ELISA experiments were performed by standard methods as described previously [3]. In brief, microtiter plates were coated with the carbohydrate antigen specified in the respective experiment (10 µg/ml in 0.04 M phosphate buffer, pH 7.0) and incubated for 18 h at 4°C. Washing steps were performed with PBS containing 0.05% Tween 20. Plates were blocked with 3% skim milk in PBS-0.02% sodium azide at 37°C for 2 h. A goat anti-rabbit IgG alkaline phosphatase conjugate (Sigma) diluted 1∶1,000 was used as secondary antibody, and *p-*nitrophenyl phosphate was used as a substrate (Sigma). After 60 min of incubation at 37°C, the absorbance was measured at 405 nm.

### Opsonophagocytic killing assay

An opsonophagocytic killing assay was used with modifications as previously described [3], [42]. In brief, *E. faecalis* strains were grown to logarithmic phase (OD 600 nm 0.4) in TSB and diluted in RPMI plus 15% heat-inactivated fetal calf serum. Baby rabbit serum (Cedarlane Laboratories, Hornby, Ontario, Canada) absorbed with the target bacterial strain served as a source of complement. White blood cells (WBC) were isolated from healthy volunteers by sedimentation with heparin-dextrane [42]. Rabbit immune serum was heat-inactivated at 56°C for 30 min before use and diluted to the concentration indicated for the individual experiments. 2.5×10^6^ white blood cells, 2.5×10^6^ CFU of *E. faecalis*, 0.00125–2.5% rabbit antiserum (as indicated in the individual experiment), and 1.7% complement in a total volume of 400 µl were incubated in tubes and rotated end over end at 37°C for 90 min. Negative controls included tubes from which leukocytes, complement or serum were omitted. The opsonic activity of the serum was calculated as follows: {1 - (CFU immune serum at 90 min/CFU of control without WBC at 90 min)} x 100. For studies on inhibition of opsonophagocytic killing, rabbit antiserum was incubated at a concentration of 1∶800 (final concentration) with various concentrations of inhibitor for 60 min at 4°C. After incubation, the opsonophagocytic killing assay was continued with the absorbed antiserum as described above. Inhibition assays were performed at serum dilutions yielding ∼70 – 80% killing of the inoculum without the addition of the inhibitor (for individual dilutions see text). The percentage of inhibition of opsonophagocytic killing was compared to controls without inhibitor.

### Mouse bacteremia model

To test protective efficacy of rabbit immune serum, we employed a modified mouse bacteremia model developed previously in our laboratory [42], [43], [44]. Six- to eight-week old female BALB-C mice (7–8 mice per group) were injected i.p. with 200 µl of heat-inactivated rabbit immune serum 48 h and 24 h before and 4 h after bacterial challenge and infected intravenously as indicated in the individual experiments. After 48 h, mice were sacrificed, blood, kidneys and livers were harvested under sterile conditions and bacterial counts enumerated by culture of serially diluted samples. The lower limit of detection of the assay was 1×10^1^ CFU.

### Ethics statement

All animal experiments were performed in compliance with the German animal protection law (TierSchG). The mice were housed and handled in accordance with good animal practice as defined by FELASA and the national animal welfare body GV-SOLAS. The animal welfare committees of the University of Freiburg (Regierungspräsidium Freiburg Az 35/9185.81/G-07/15) approved all animal experiments. The institutional review board of the University of Freiburg approved the study protocol and written informed consent was obtained from all study participants.

### Statistcal analysis

Statistical significance for two-way comparisons was determined by an unpaired t test. Analysis of variance (ANOVA) for multigroup comparisons was used on log-transformed data, and the Tukey's multiple-comparison test was used for posthoc analysis for pairwise comparisons. Statistical results were calculated using the Prism 3 software package.

## Supporting Information

Table S1 ^1^H and ^13^C NMR data of the capsular polysaccharide from *E. faecalis* strain type 2. Spectra were recorded of a solution in ^2^H_2_O at 600 MHz and 27°C relative to internal acetone (δ_H_ 2.225; δ_C_ 31.45).
(DOC)

Table S2 ^1^H and ^13^C NMR chemical shifts [δ] of diheteroglycan isolated from of *E. faecalis* type 5. Spectra were recorded of a solution in ^2^H_2_O at 600 MHz and 27°C relative to internal acetone (δ_H_ 2.225; δ_C_ 31.45).
(DOC)

Figure S1 SDS PAGE electrophoresis of purified diheteroglycan from *E. faecalis* type 2. Lane 1 protein molecular mass marker, lane 2 Coomassie stain, lane 3 PAS stain.
(TIFF)

Figure S2 Section of the HMBC spectrum of diheteroglycan isolated from *E. faecalis* type 2. The *inter*residual connectivities are underlined.
(PDF)

Figure S3 The ^1^H NMR spectrum of diheteroglycan isolated from: **A** *E. faecalis* type 5, **B** *E. faecalis* FA2-2. The *letters* refer to the carbohydrate residues as shown in chemical structure (Fig. 2C), and the *arabic numbers* refer to the protons in the respective residues; LA, lactic acid. * Acetic acid remainder of the final gel-permeation chromatography step.
(PDF)

Figure S4 Section of the ROESY spectrum of diheteroglycan isolated from *E. faecalis* type 5. The spectrum was recorded at 600 MHz and 27°C. The *inter*residual NOE contacts are underlined.
(PDF)

Figure S5 ELISA of rabbit antiserum raised against purified diheteroglycan from *E. faecalis* type 2 and type 5. Microtiter plates were coated with the respective polysaccharide (1 µg/well) and incubated with serum dilutions of immune rabbit serum against the homolgous strain as indicated in the graph.
(TIFF)
