# Supplementary material for: Serodiversity of Opsonic Antibodies against Enterococcus faecalis —Glycans of the Cell Wall Revisited
Source: PLoS One. 2011 Mar 18;6(3):e17839. doi: 10.1371/journal.pone.0017839 (PMC3060912; doi:10.1371/journal.pone.0017839)
Supplement: Table S1 — 1H and 13C NMR data of the capsular polysaccharide from E. faecalis strain type 2. Spectra were recorded of a solution in 2H2O at 600 MHz and 27°C relative to internal acetone (δH 2.225; δC 31.45). (DOC) [file pone.0017839.s001.doc]

299(5615):2071-2074.

| **Strain** | **Serotype** | **Characterization** | **Reference** |
| --- | --- | --- | --- |
| *E. faecalis* 12030 | CPS-A | Clinical isolate | (18) |
| *E. faecalis* 12107 | CPS-B | Clinical isolate | (47) |
| *E. faecalis* OG1RF | CPS-B | Laboratory strain ATCC 47077 | (48) |
| *E. faecalis* type 1 | CPS-B | Protype strain | (31) |
| *E. faecalis* type 2 | CPS-C | Protype strain | (31) |
| *E. faecalis* type 21 | CPS-C | Protype strain | (31) |
| *E. faecalis* R19.001 | CPS-C | Epidemic strain | (49) |
| *E. faecalis* V583 | CPS-C | Vancomycin resistant strain, fully sequenced ATCC 700802 | (50) |
| *E. faecalis* type FA-2-2 | CPS-C | Laboratory strain | (11) |
| *E. faecalis* type HG101 | -- | *cpsI* mutant of FA2-2 | (11) |
| *E. faecalis* type 5 | CPS-D | Protype strain | (31) |

|  |  | | | | | | |
| --- | --- | --- | --- | --- | --- | --- | --- |
|  |  |  |  |  |  |  |
|  |  |  |  |  |  |  |  |
|  |  |  |  |  |  |  |  |
|  |  |  |  |  |  |  |  |
|  |  |  |  |  |  |  |  |
