# Supplementary material for: Serodiversity of Opsonic Antibodies against Enterococcus faecalis —Glycans of the Cell Wall Revisited
Source: PLoS One. 2011 Mar 18;6(3):e17839. doi: 10.1371/journal.pone.0017839 (PMC3060912; doi:10.1371/journal.pone.0017839)
Supplement: Table S2 — 1H and 13C NMR chemical shifts [δ] of diheteroglycan isolated from of E. faecalis type 5. Spectra were recorded of a solution in 2H2O at 600 MHz and 27°C relative to internal acetone (δH 2.225; δC 31.45). (DOC) [file pone.0017839.s002.doc]

|  |  | | | | | | | | | | | | | |  |
| --- | --- | --- | --- | --- | --- | --- | --- | --- | --- | --- | --- | --- | --- | --- | --- |
|  |  |  |  |  |  |  |  |  |  |  |  |  |  |  |  |
|  |  | |  | |  | |  | |  | |  | |  | |  |
|  |  | |  | |  | |  | |  | |  | |  | |  |
|  |  | |  | |  | |  | |  | |  | |  | |  |
|  |  | |  | |  | |  | |  | |  | |  | |  |
| **Residue** | | **Chemical shifts ^1^H and ^13^C [δ]** | | | | | | | | | | | | | |
|  |  | H1  C1 | | H2 C2 | | H3  C3 | | H4  C4 | | H5  C5 | | H6*^a^*  C6 | | H6*^b^* | |
| →36)-β-Gal*f*-  A | | 5.315  109.26 | | 4.346  80.26 | | 3.932  84.96 | | 4.225  82.41 | | 4.040  70.55 | | 3.768  71.90 | | 4.019 | |
| →3)-β-D-Glc*p*-  B | | 4.542  103.22 | | 3.460  74.05 | | 3.663  82.44 | | 3.465  68.8 | | 3.500  76.26 | | 3.742  61.26 | | 3.930 | |
| Lactic acid  LA | | 181.29 | | 4.038  77.72 | | 1.361  19.21 | |  | |  | |  | |  | |
