# Supplementary figures and images for: Serodiversity of Opsonic Antibodies against Enterococcus faecalis —Glycans of the Cell Wall Revisited
Source: PLoS One. 2011 Mar 18;6(3):e17839. doi: 10.1371/journal.pone.0017839 (PMC3060912; doi:10.1371/journal.pone.0017839)

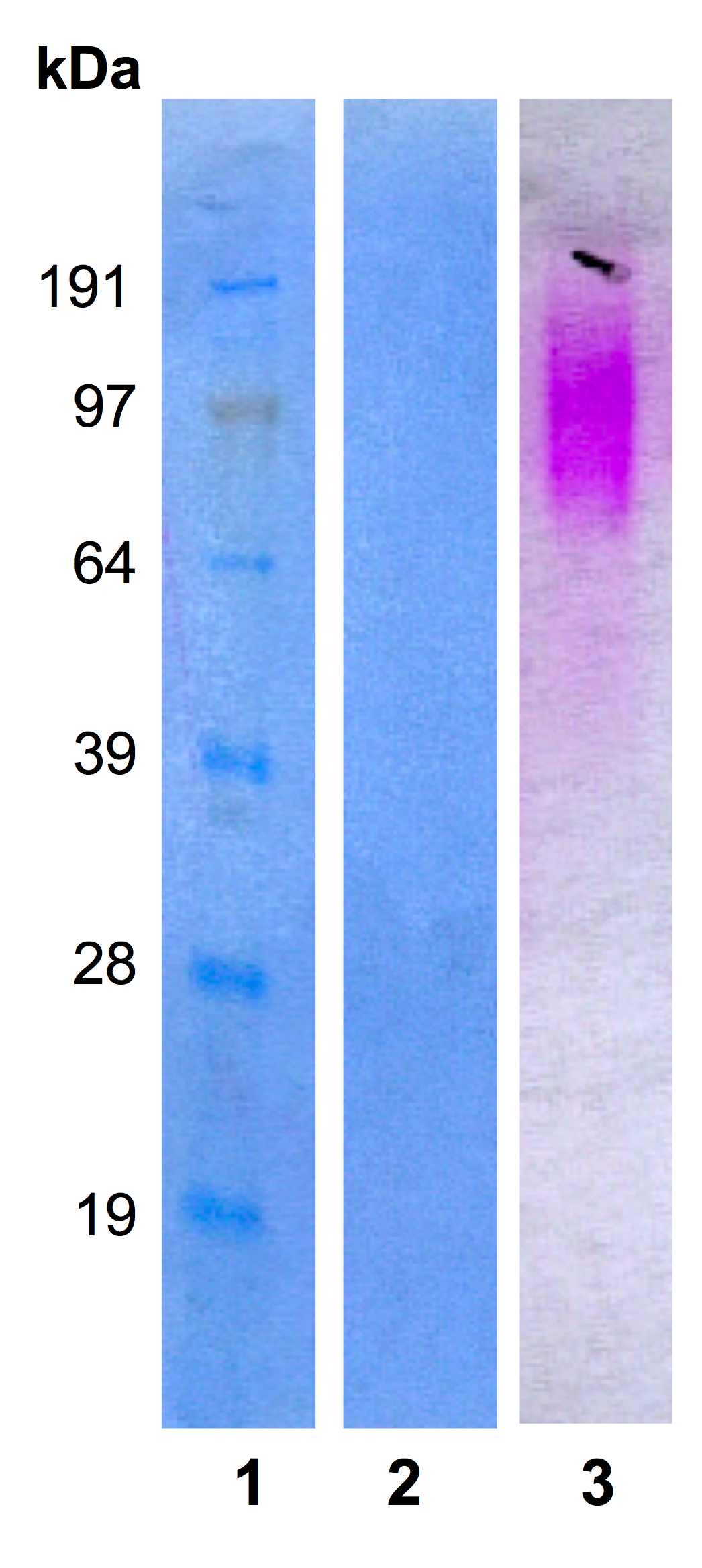

Supplement: Figure S1 — SDS PAGE electrophoresis of purified diheteroglycan from E. faecalis type 2. Lane 1 protein molecular mass marker, lane 2 Coomassie stain, lane 3 PAS stain. (TIFF) [file pone.0017839.s003.tif]

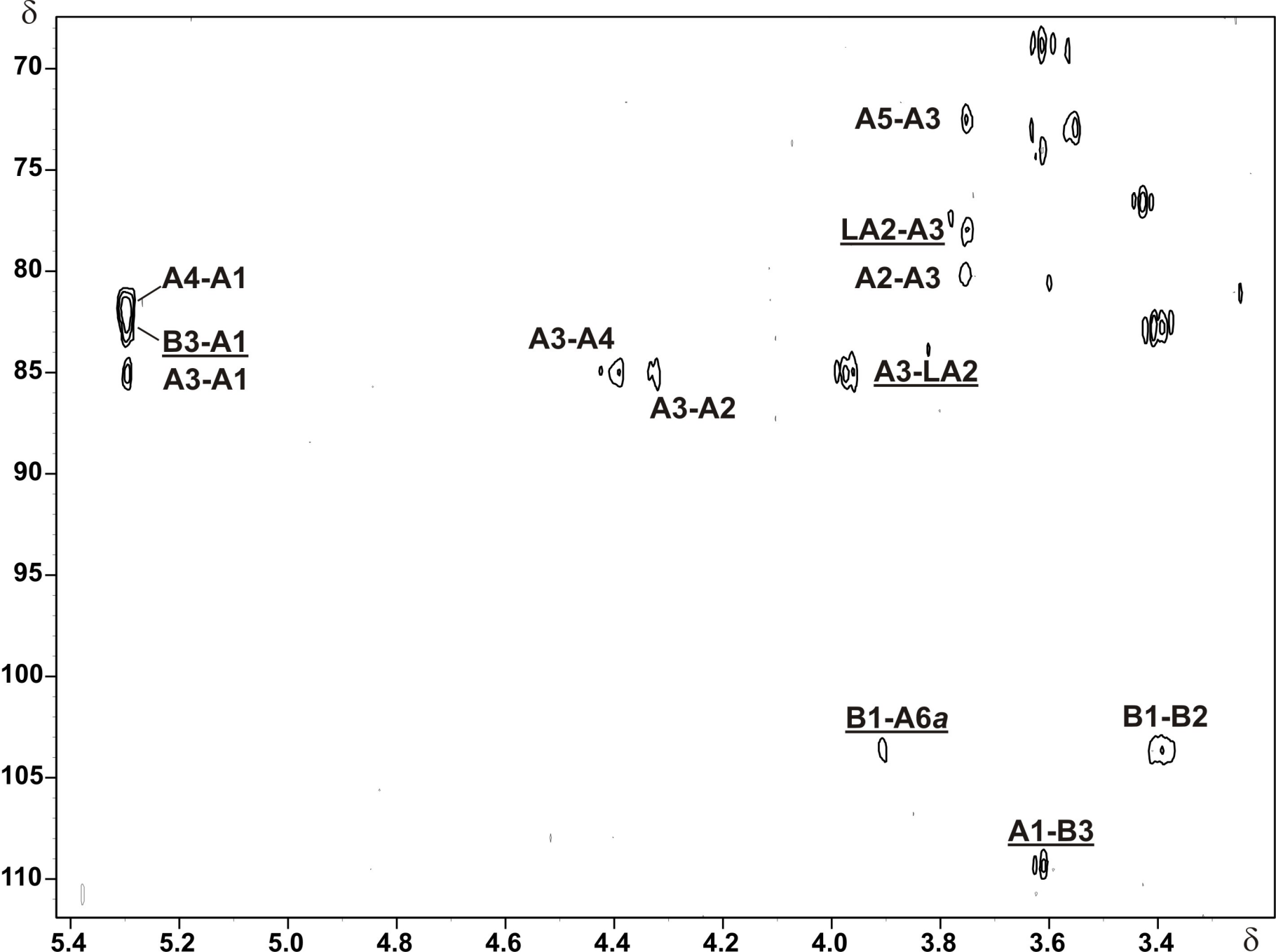

Supplement: Figure S2 — Section of the HMBC spectrum of diheteroglycan isolated from E. faecalis type 2. The interresidual connectivities are underlined. (PDF) [file pone.0017839.s004.pdf]

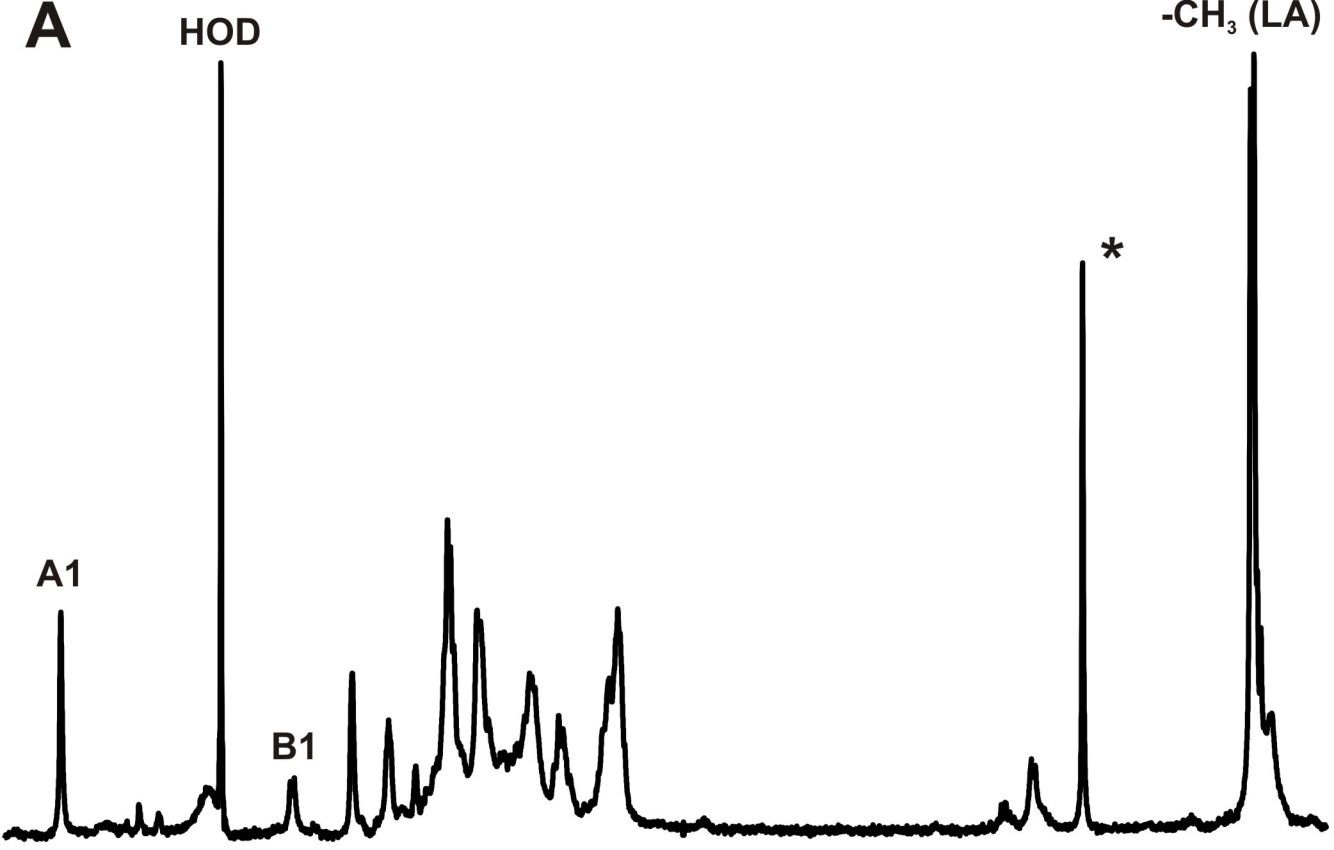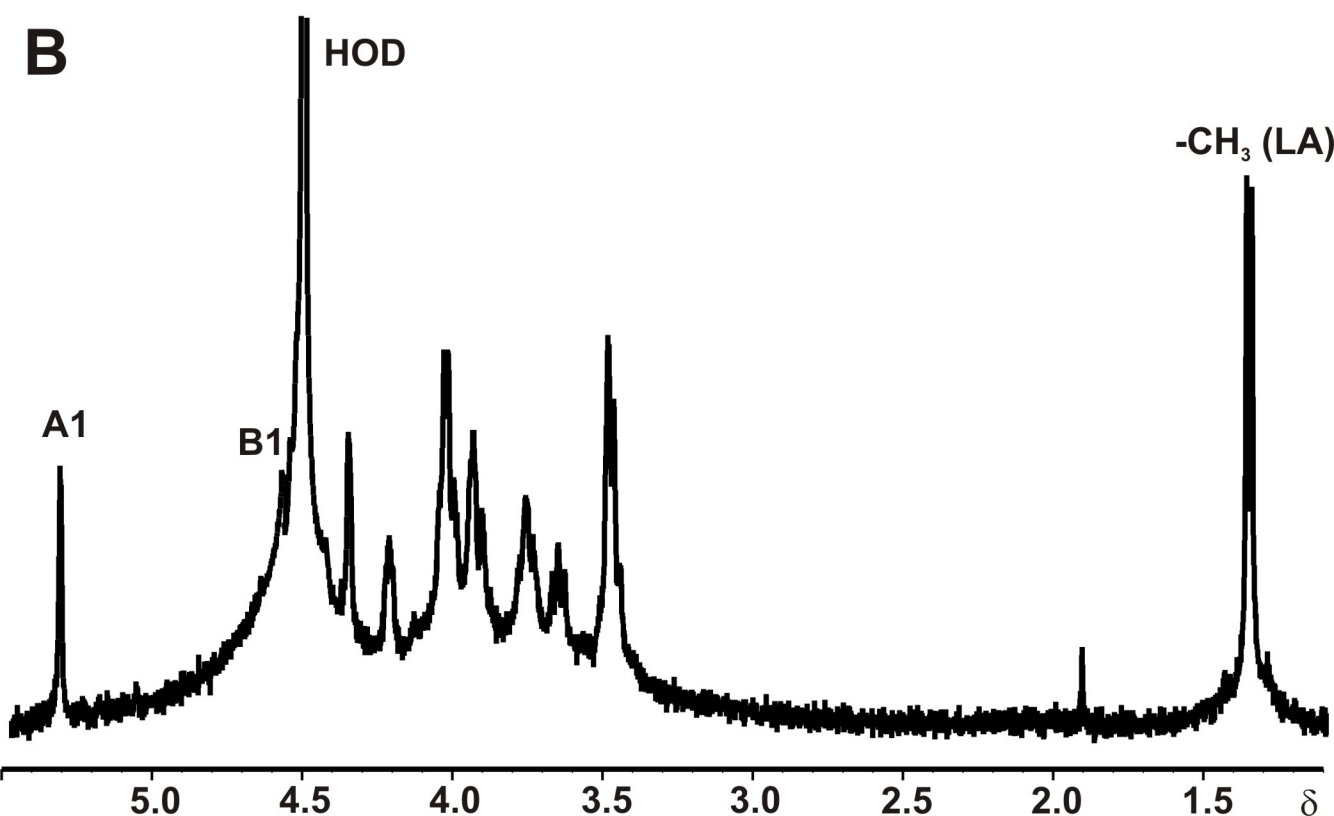

Supplement: Figure S3 — The 1H NMR spectrum of diheteroglycan isolated from: A E. faecalis type 5, B E. faecalis FA2-2. The letters refer to the carbohydrate residues as shown in chemical structure (Fig. 2C), and the arabic numbers refer to the protons in the respective residues; LA, lactic acid. * Acetic acid remainder of the final gel-permeation chromatography step. (PDF) [file pone.0017839.s005.pdf]

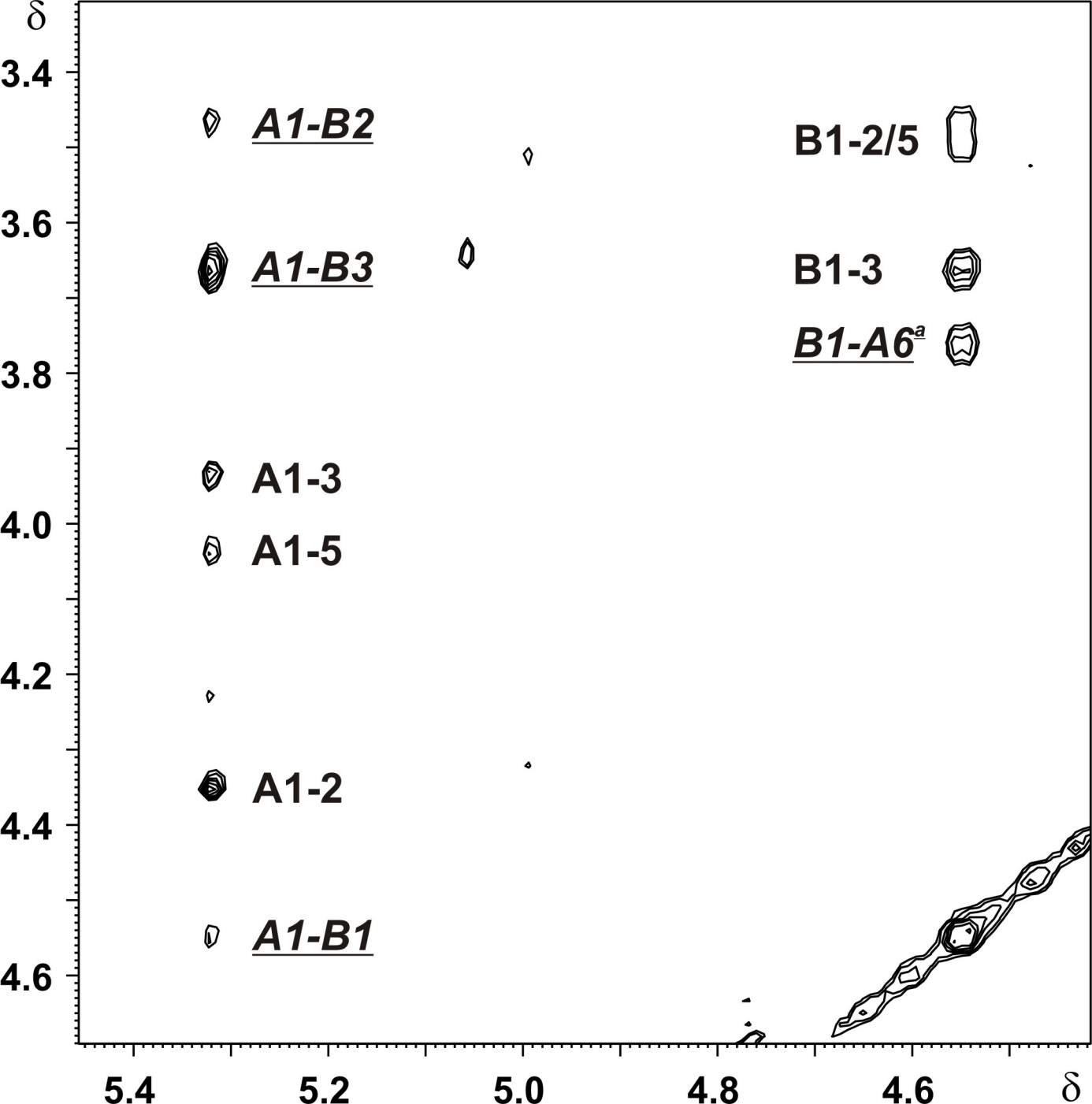

Supplement: Figure S4 — Section of the ROESY spectrum of diheteroglycan isolated from E. faecalis type 5. The spectrum was recorded at 600 MHz and 27°C. The interresidual NOE contacts are underlined. (PDF) [file pone.0017839.s006.pdf]

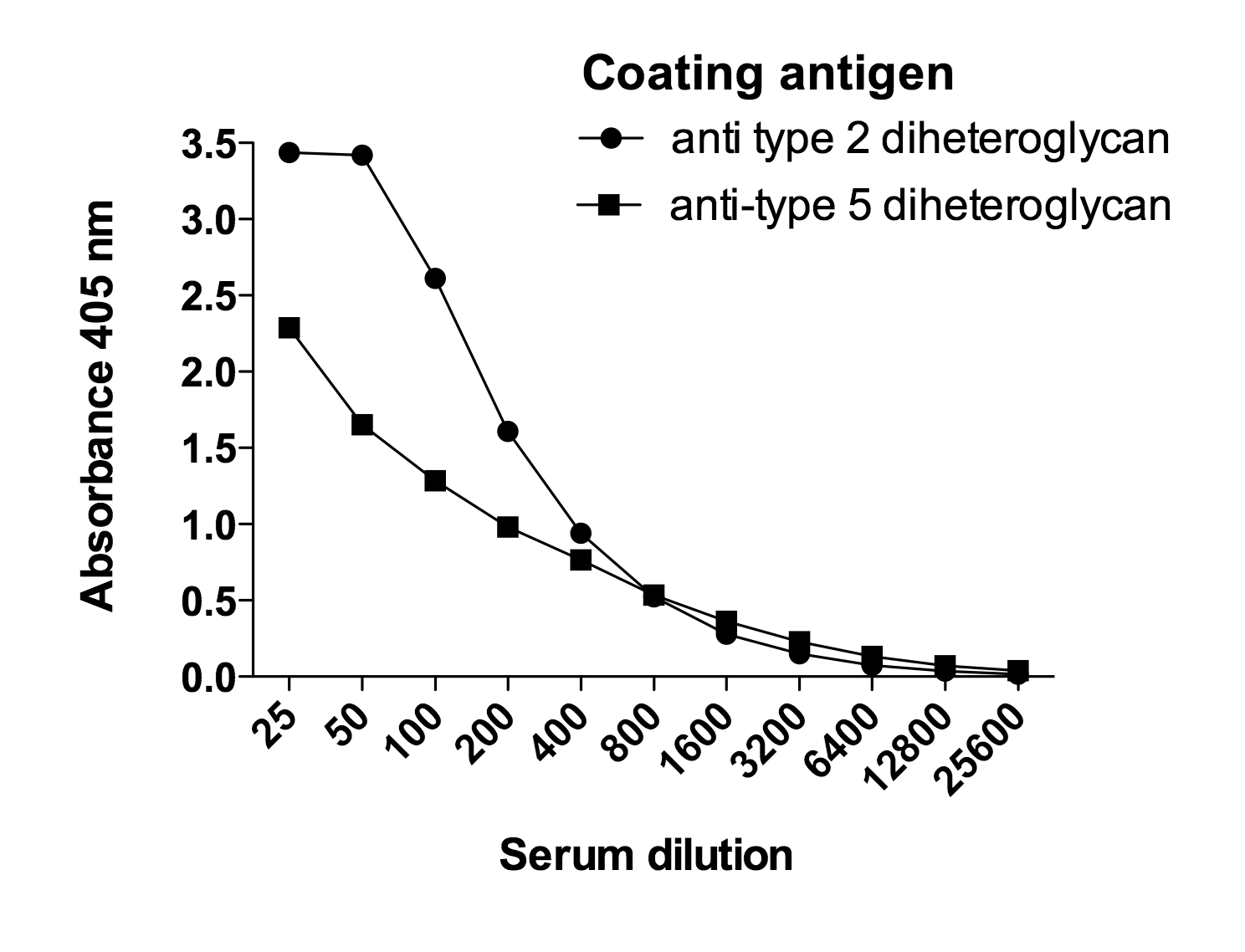

Supplement: Figure S5 — ELISA of rabbit antiserum raised against purified diheteroglycan from E. faecalis type 2 and type 5. Microtiter plates were coated with the respective polysaccharide (1 µg/well) and incubated with serum dilutions of immune rabbit serum against the homolgous strain as indicated in the graph. (TIFF) [file pone.0017839.s007.tif]
